# 551. Efficacy of adjusting steroids dose in non-ICU COVID-19 pneumonia patients with chest radiology deterioration

**DOI:** 10.1093/ofid/ofad500.620

**Published:** 2023-11-27

**Authors:** Qingqing Wang, Bijie Hu

**Affiliations:** Zhongshan hospital, Fudan University, Shanghai, Shanghai, China; Department of Infectious Diseases, Zhongshan Hospital, Fudan University, Shanghai, Shanghai, China

## Abstract

**Background:**

Steroids is an effective medicine to improve lung lesions and clinical prognosis in COVID-19 pneumonia patients. The outcomes and chest CT images of steroid dose adjustment in CT progressive patients is still unknown. The study is to investigate the effects of increasing steroids dosage to chest CT areas and clinical outcomes in non-severe COVID-19 patients with progressive chest CT lesions.

**Figure 1.**
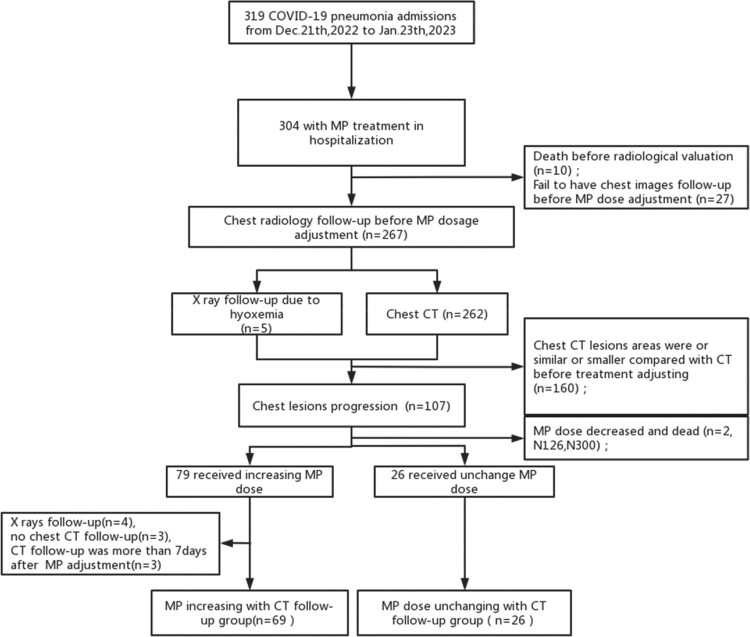
Chart flow

**Methods:**

This retrospective study used Zhongshan hospital affiliated to Fudan University clinial data to identify 319 adult hospitalized in infectious diseases department for COVID-19 pneumonia between December 2022 and January 2023. We made a treatment strategy tailoring the steroids dosage according to the CT chest images to optimizing methylprednisolone (MP) dose precisely in these patients. Chest radiology were performed at admission and were follow-up during MP treatment. According to adjustment of MP dosage, we classified patients with radiology progression into MP increment group and MP no-change group. Clinical features, CT severity scores changes within 7 days after steroids adjustment, 96 hours WHO outcome scores, length of stay (LOS) and 28-day mortality rate were recorded and assessed between groups.

**Results:**

Among 319 participants, average age were 72(IQR 66-82), male were 207(64.9%), severe illness accounted for 44.2%(n=141). 105 patients with aggressive chest radiology during MP treatment were classified into increment group(n=79) and no-change group(n=26). 6(7.6%) and 1(3.8%) patients had increasing WHO outcome scores 96h after MP adjustment(p=0.678), LOS [15(IQR 10-24) vs 14(IQR 10-25) (p=0.994)] and 28-day mortality (7.6% vs 3.8%, p=0.678) showed no significant differences in increment group and no-change group respectively. 95 patients have available chest CT follow-up with 7 days after CT progressive. Meanwhile, univariate and multivariate logistic regressive analysis revealed that patients receiving increased MP dose was significantly associated with improvement in CT lesion areas compared with those in no-change group(79.7% vs 53.8%, p=0.044).

**Figure 2.**
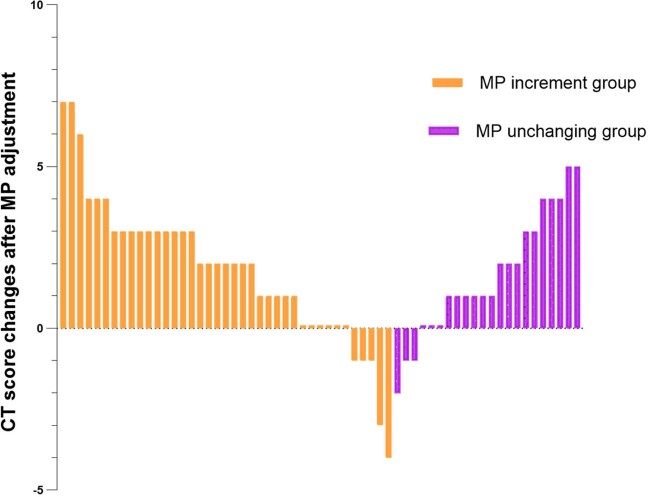
Chest CT score changes after MP dose adjustment in two groups.

The orange and purple bars present CT score changes in MP increment group and in MP unchanging group respectively. These CT score changes showed no significant differences in groups.

**Figure 2.**
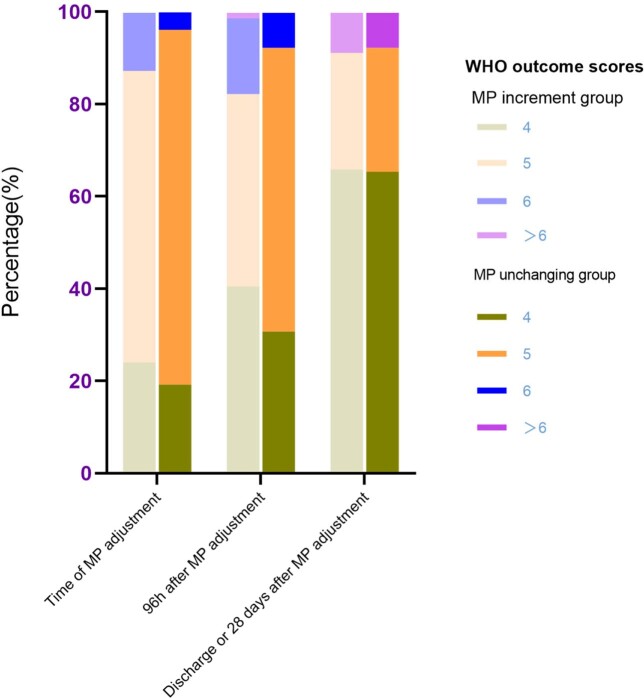
The comparison of WHO outcome scores proportion and dynamics in groups.

The bar with light color presents MP increment group and the bar with dark color presents MP unchanging group. the three sets of bars showed the dynamics of WHO outcome scores components before and after MP dose adjustment.

**Figure 3.**
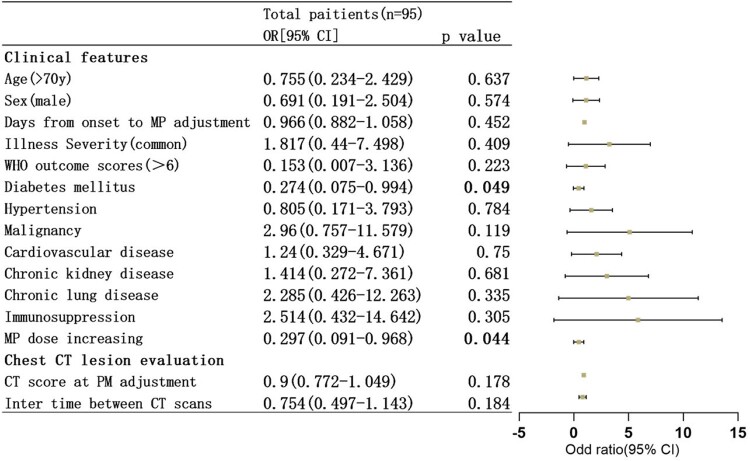
Multivariate logistic analysis of clinical and CT features for non- ICU COVID-19 pneumonia patients with lung lesions improvement.

Odd ratios are plotted as squares, with the size of each square proportional to the amount of statistical information that was available; the horizontal lines represent 95% confidence intervals.

**Conclusion:**

Increasing MP dosage in CT worsening patients improves CT lesions while fails to decrease serious adverse outcomes.

**Disclosures:**

**All Authors**: No reported disclosures

